# Hyperactive Delirium Temporally Associated With Hydroxyzine in a Child With Succinic Semialdehyde Dehydrogenase Deficiency Presenting With Psychotic Symptoms: A Case Report

**DOI:** 10.1155/crps/3543154

**Published:** 2026-10-08

**Authors:** Musa Yilanli, Colton Olexa, Bianca Zapanta

**Affiliations:** ^1^ Department of Psychiatry and Behavioral Health Nationwide Children’s Hospital Columbus Ohio USA nationwidechildrens.org; ^2^ Department of Psychiatry and Behavioral Health The Ohio State University Columbus Ohio USA osu.edu; ^3^ Center for Biobehavioral Health Nationwide Children’s Hospital Columbus Ohio USA nationwidechildrens.org; ^4^ Division of Genetic and Genomic Medicine Nationwide Children’s Hospital Columbus Ohio USA nationwidechildrens.org; ^5^ Department of Pediatrics The Ohio State University College of Medicine Columbus Ohio USA osu.edu

**Keywords:** hydroxyzine, hyperactive delirium, metabolic disorder, pediatric psychiatry, psychotic symptoms, quetiapine, SSADHD, succinic semialdehyde dehydrogenase deficiency

## Abstract

**Background:**

Succinic semialdehyde dehydrogenase deficiency (SSADHD) is a rare autosomal recessive disorder of gamma‐aminobutyric acid (GABA) metabolism caused by biallelic variants in ALDH5A1. The phenotype includes developmental delay, intellectual disability, seizures, sleep disturbance, anxiety, and other neurobehavioral symptoms. Psychotic symptoms are increasingly recognized in SSADHD but remain uncommon and incompletely characterized in children. In medically and neurodevelopmentally vulnerable patients, acute psychiatric decompensation may also be complicated by delirium, especially when severe sleep–wake disruption, poor intake, constipation, metabolic stress, and potentially deliriogenic medications are present.

**Case Presentation:**

We describe a 12‐year‐old girl with intellectual disability, global developmental delay, abnormal prior neuroimaging, abnormal EEG findings, progressive weight loss, reduced oral intake, insomnia, and new‐onset auditory and visual hallucinations. During hospitalization, she developed a fluctuating neurobehavioral syndrome marked by impaired attention, severe sleep–wake disruption, increased startle response, inconsolability, agitation, perceptual disturbances, and waxing and waning behavioral organization. These findings supported hyperactive delirium superimposed on psychotic symptoms rather than isolated psychosis. Hydroxyzine was ordered as 25 mg by mouth every 6 h as needed and was administered six times from hospital day 5 through early hospital day 7. The medication provided mixed short‐term anxiolytic and sedating benefits but did not meaningfully reduce hallucinations or restore normal sleep. After hydroxyzine exposure, impaired attention, profound sleep–wake dysregulation, inconsolability, increased startle response, fluctuating agitation, and behavioral disorganization remained prominent, raising concern that hydroxyzine may have contributed to persistence or worsening of the acute confusional state in the setting of severe sleep deprivation, poor oral intake, constipation, neurologic vulnerability, and underlying SSADHD. Hydroxyzine was discontinued, and low‐dose quetiapine was initiated together with environmental sleep–wake and reorientation interventions. Sleep, appetite, oral intake, and behavioral regulation subsequently improved. Diagnostic evaluation supported SSADHD, including elevated urinary 4‐hydroxybutyric acid, characteristic MRI abnormalities of the globi pallidi and dentate nuclei, and a homozygous ALDH5A1 missense variant, NM_001080.3:c.434A >G (p.Glu145Gly), formally classified as a variant of uncertain significance but considered clinically concordant with the diagnosis.

**Conclusion:**

This case highlights SSADHD as an important consideration in children presenting with acute psychotic symptoms and altered mental status. It also underscores the importance of distinguishing psychotic symptoms from superimposed delirium in developmentally vulnerable patients. Because hallucinations, insomnia, poor intake, and behavioral dysregulation preceded hydroxyzine exposure and several competing contributors were present, hydroxyzine should be framed as a possible exacerbating or prolonging factor rather than the definitive sole cause of delirium. The case is therefore best described as hyperactive delirium temporally associated with, and possibly exacerbated by, hydroxyzine exposure.

**Summary**

• Succinic semialdehyde dehydrogenase deficiency (SSADHD) may present with acute psychotic symptoms during adolescence, particularly in the context of developmental and neurologic vulnerability.
• Hydroxyzine exposure was temporally associated with persistent and clinically apparent hyperactive delirium features, but causality is best classified as possible rather than definite.
• Careful differentiation of psychosis from delirium, medication reconciliation, sleep restoration, and multidisciplinary management were central to stabilization.

## 1. Introduction

Succinic semialdehyde dehydrogenase deficiency (SSADHD) is a rare inherited neurometabolic disorder caused by deficiency of succinic semialdehyde dehydrogenase, the enzyme encoded by the ALDH5A1 gene [1, 2]. Disruption of the gamma‐aminobutyric acid (GABA) degradation pathway leads to accumulation of gamma‐hydroxybutyrate (GHB) and related metabolic abnormalities. The clinical phenotype is heterogeneous and commonly includes developmental delay, intellectual disability, speech impairment, hypotonia, seizures, ataxia, sleep disturbance, anxiety, hyperkinetic behavior, aggression, and other neurobehavioral symptoms [1–3].

Psychotic symptoms occur in a minority of patients with SSADHD. A qualitative review found that psychosis most often presents as auditory or visual hallucinations, with onset typically in adolescence or young adulthood, although childhood cases have been reported [4]. Published reports also describe severe anxiety, behavioral dysregulation, aggression, sleep disturbance, catatonia‐like features, and response to cautiously selected antipsychotic treatment in some patients [4–6]. Because developmental and language limitations can complicate symptom reporting, psychosis in SSADHD may be underrecognized or misattributed to a primary psychiatric disorder.

Delirium adds further diagnostic complexity. Pediatric delirium is characterized by an acute change from baseline with disturbance in attention and awareness, fluctuation, altered arousal or behavioral organization, perceptual disturbance, and sleep–wake disruption [7]. These features may overlap with psychosis, especially in children with intellectual disability, neurologic abnormalities, sleep deprivation, poor oral intake, constipation, or medication exposure. We report a child with SSADHD who presented with acute psychotic symptoms and developed hyperactive delirium features during hospitalization. Hydroxyzine is discussed as a possible exacerbating or prolonging contributor, not as a proven sole cause, because substantial neuropsychiatric symptoms were present before exposure and multiple competing etiologies remained plausible.

## 2. Case Presentation

### 2.1. Patient Background

The patient was a 12‐year‐old girl with an intellectual disability, global developmental delay, speech delay, anemia, and abnormal prior neurodiagnostic findings. She was admitted on HD1 and discharged on HD10. The admission weight was 50.7 kg.

At ~3 years of age, she underwent evaluation for developmental delay, language regression, toe walking, and abnormal brain MRI findings concerning a metabolic disorder. Additional metabolic testing was recommended in early childhood but was not completed because of difficulty tolerating blood draws and subsequent loss to follow‐up. There was no prior formal psychiatric treatment and no documented preadmission psychotropic or antiseizure medication. Family history was notable for parental consanguinity, an intellectual disability in a maternal relative, and psychotic illness in paternal relatives.

During the month before admission, she developed progressive decreased oral intake, unintentional 15‐lb weight loss, worsening insomnia, anxiety, and fearfulness, and new‐onset hallucinatory and paranoid symptoms. Her parents reported statements such as, “I am scared,” descriptions of seeing ghosts or a threatening figure, concern that someone was coming to get her, and repeated references to a former teacher. The symptoms occurred during a difficult transition from elementary school to middle school, but their severity, associated weight loss, and perceptual disturbances prompted medical admission and multidisciplinary evaluation. The clinical course, including symptom evolution, diagnostic evaluation, medication exposure, and treatment response, is summarized by hospital day in Table 1.

**Table 1:** Clinical timeline.

Hospital day	Clinical features and work‐up	Medication/interventions	Outcome
Preadmission, ~1 month	Progressive insomnia, anxiety, fearfulness, decreased oral intake, 15‐lb weight loss, and new auditory/visual hallucinations with paranoid content.	No documented preadmission psychotropic or antiseizure medication. Family pursued medical care because of worsening function and intake.	Acute change from developmental baseline before hydroxyzine exposure.
HD1 (hospital day 1)	Admission for weight loss, poor intake, insomnia, behavioral change, paranoia, and hallucinations. Head CT negative. Abdominal radiography showed pancolonic stool burden.	IV fluids and bowel regimen initiated. No standing psychotropic medication.	Medical, neurologic, psychiatric, psychological, and genetic evaluation initiated.
HD2–HD4	Ongoing fearfulness, poor intake, impaired attention, perseveration, hallucinations, and behavioral dysregulation. Catatonia considered but not supported as primary explanation.	Melatonin used for sleep; bowel regimen continued. No antipsychotic initially while diagnostic work‐up continued.	Symptoms remained severe and fluctuating.
HD5	Marked agitation, paranoia, crying, fearfulness, and hallucinations; impaired attention and poor redirection documented before repeated hydroxyzine exposure.	Hydroxyzine initiated/ordered as 25 mg PO every 6 h PRN. Two liquid doses of 26 mg were administered on HD5.	Partial short‐term calming/sleep effect, but hallucinations and sleep disturbance persisted.
HD6	Ongoing hallucinations, paranoia, insomnia, impaired attention, increased startle response, inconsolability, and disrupted day–night cycle. Delirium risk explicitly documented.	Two 25 mg PO hydroxyzine doses were administered on HD6. Quetiapine was discussed but initially declined by the family.	Hydroxyzine did not meaningfully improve hallucinations or normalize sleep.
HD7	Severe fluctuating distress, crying, yelling, fearfulness, repeated statements of distress, report of seeing “a man,” impaired attention, and waxing/waning behavioral organization. MRI compatible with suspected SSADHD. LTM EEG abnormal but without seizures.	Two 25 mg PO hydroxyzine doses were administered early on HD7, then hydroxyzine was discontinued because of limited efficacy and concern for anticholinergic burden. Quetiapine 25 mg PO nightly was started that evening; melatonin 5 mg was also given.	Delirium formulated as hyperactive delirium due to multiple etiologies, with hydroxyzine as a possible contributor.
HD8	Parents reported best sleep since admission and improved appetite. Continued hallucinations and distress occurred, but she had more periods of calm and age‐appropriate engagement.	A daytime quetiapine 12.5 mg PO PRN dose and a quetiapine 25 mg PO nightly dose were administered. Environmental delirium interventions continued.	Improved sleep, appetite, oral intake, and behavioral regulation.
HD9	Symptoms continued to fluctuate, with intermittent fearfulness and hallucinations but increasing periods of play, dancing, bathing, family interaction, and broader affect.	Quetiapine 25 mg PO nightly; melatonin 5 mg nightly; sleep–wake regulation and reorientation supports.	Less prolonged dysregulation and improved participation in preferred activities.
HD10	Improved sleep quality, oral intake, redirectability, and calm intervals; intermittent hallucinations had not fully resolved but were less continuously impairing.	Discharged on quetiapine 25 mg nightly with outpatient psychiatry and genetics follow‐up.	Partial resolution of hyperactive delirium syndrome and improved clinical stability.

The patient weighed 50.7 kg at admission; hydroxyzine 25 mg was ~0.49 mg/kg/dose, and hydroxyzine 26 mg was ~0.51 mg/kg/dose. Quetiapine 25 mg was ~0.49 mg/kg/dose and quetiapine 12.5 mg was ~0.25 mg/kg/dose. Medication exposures and administered doses during hospitalization are summarized in Table 2.

**Table 2:** Medication administration summary.

Medication	Dose, route, and hospital‐day administrations	Indication/comment
Hydroxyzine HCl oral liquid	Ordered as 25 mg PO every 6 h PRN. Administered six times from HD5 through early HD7: 26 mg twice on HD5, 25 mg twice on HD6, and 25 mg twice early on HD7. Total administered dose: 152 mg over HD5 to early HD7.	Used for anxiety, agitation, and sleep disturbance. Provided mixed short‐term benefit but did not meaningfully improve hallucinations or restore normal sleep. Discontinued on HD7 because of limited efficacy and concern for anticholinergic burden in delirium.
Quetiapine oral suspension	25 mg PO nightly was initiated on HD7; 12.5 mg PO PRN was given during the day on HD8; 25 mg PO nightly was continued on HD8 and HD9.	Initiated after hydroxyzine discontinuation to target sleep–wake dysregulation, agitation, psychotic symptoms, and hyperactive delirium features.
Melatonin oral liquid	5 mg PO was administered during HD4 and then nightly on HD7–HD9.	Used to support sleep–wake regulation.
Bowel regimen	Polyethylene glycol 17 g PO was administered multiple times across HD2–HD4, then PRN; sennosides 12 mg PO was administered on HD3–HD4.	Treatment of pancolonic stool burden/constipation, a potential delirium contributor.
IV fluids	Dextrose 5%‐lactated Ringer’s with KCl 20 mEq/L at 90 mL/h was administered on HD2; lactated Ringer’s at 90 mL/h was administered on HD2–HD3 and HD7.	Supportive treatment for poor intake/dehydration risk.
Acetaminophen	650–768 mg PO PRN was administered during HD2 and again during HD6–HD7.	Analgesia/comfort; no opioid or benzodiazepine exposure was documented in the available medication administration record.
Pediatric multivitamin with iron	One chewable tablet PO daily during admission when tolerated.	Nutritional support in the setting of anemia and poor intake.

### 2.2. Investigations and Rule‐Out Work‐Up

Initial laboratory evaluation was notable for mild microcytic anemia and a low serum bicarbonate level with mild anion gap elevation; however, the available studies did not identify a single alternative medical etiology sufficient to explain the acute neuropsychiatric syndrome. Admission laboratory values included sodium 138 mmol/L, potassium 3.7 mmol/L, chloride 106 mmol/L, bicarbonate 18 mmol/L, blood urea nitrogen 9 mg/dL, creatinine 0.40 mg/dL, glucose 122 mg/dL, calcium 9.8 mg/dL, alanine aminotransferase 8 U/L, aspartate aminotransferase 22 U/L, alkaline phosphatase 240 U/L, total bilirubin 0.8 mg/dL, lipase 88 U/L, and ammonia <9 µmol/L. A complete blood count demonstrated a white blood cell count of 8.9 × 10^3^/µL, hemoglobin of 10.7 g/dL, hematocrit of 33.3%, platelet count of 442 × 10^3^/µL, and mean corpuscular volume of 66.6 fL. Inflammatory and endocrine markers were not suggestive of an alternative systemic process, with an erythrocyte sedimentation rate of 11 mm/h, C‐reactive protein <0.5 mg/dL, free thyroxine of 1.2 ng/dL, and thyroid‐stimulating hormone of 2.101 µIU/mL. Venous blood gas obtained on hospital day 2 showed pH 7.39, pCO_2_ 34 mmHg, bicarbonate 20 mmol/L, and base deficit 4.7; lactate was 2.1 mmol/L.

During the period of concern for delirium, the patient remained afebrile and had stable oxygenation when recorded. Vital signs during the peak symptom period included blood pressure ranging from 115/68 to 122/69 mmHg, heart rate of 73–89 beats/min, temperature of 36.1 to 36.4°C, respiratory rate of 14–16 breaths/min, and an oxygen saturation of 99%. Urine drug screening was negative, and heavy metal testing, including arsenic, lead, and mercury, was unrevealing. Urinalysis did not support urinary tract infection, and serum human chorionic gonadotropin testing was negative. Head computed tomography did not show an acute intracranial abnormality. Abdominal radiography demonstrated a pancolonic stool burden, which was treated with a bowel regimen and considered a potential contributing vulnerability rather than a primary explanation for the full neuropsychiatric presentation. The diagnostic evaluation for medical, neurologic, metabolic, toxicologic, infectious, and other potential contributors to the acute neuropsychiatric presentation is summarized in Table 3.

**Table 3:** Summary of diagnostic investigations.

Domain	Findings and interpretation
Basic laboratory evaluation	Mild microcytic anemia; low bicarbonate with mild anion gap elevation early in admission; glucose, renal function, liver enzymes, ammonia, ESR, CRP, TSH, and free T4 did not identify an alternative unifying cause.
Toxicology and exposure testing	Urine drug screen negative. Heavy metals negative: arsenic less than 10.0 µg/L, lead less than 2.0 µg/dL, mercury less than 2.5 µg/L.
Infectious/inflammatory assessment	Afebrile course; WBC and inflammatory markers not suggestive of acute infection. HIV negative and RPR nonreactive. No CSF studies were available.
Gastrointestinal/nutritional factors	Abdominal radiography demonstrated pancolonic stool burden; poor intake and weight loss were clinically significant contributors to vulnerability.
EEG/seizure evaluation	Long‐term video EEG over HD5–HD6 was abnormal for age, with bifrontal spike‐wave discharges, rhythmic delta activity, and intermittent sharply contoured left frontal theta activity considered potentially epileptogenic. No electrographic seizures were recorded, reducing concern for nonconvulsive status during monitoring.
Brain imaging	MRI on HD7 demonstrated T2 hyperintensity in the bilateral globi pallidi and dentate nuclei, prominent perivascular spaces, and mineral deposition within the globus pallidus, without abnormal enhancement or restricted diffusion. Findings were compatible with suspected SSADHD.
Metabolic testing	Urine organic acids demonstrated an increased 4‐hydroxybutyric acid peak most consistent with SSADHD, with increased lactate also reported.
Autoimmune/first‐episode psychosis work‐up	Copper 113 µg/dL, ceruloplasmin 23 mg/dL, vitamin B1 72 nmol/L, cortisol 9.0 µg/dL, HIV negative, RPR nonreactive. ANA was positive at 1:160 and GAD65 antibody was low‐positive at 0.04 nmol/L; broader pediatric autoimmune CNS panel was otherwise negative, including NMDA receptor, CASPR2, LGI1, GABA‐B receptor, GFAP, MOG, and NMO/AQP4 antibodies.
Cardiac monitoring	ECG on HD5 showed heart rate 103 beats/min, PR interval 136 ms, QRS duration 78 ms, QT/QTc 316/418 ms, without clinically significant QTc prolongation.

### 2.3. Genetic and Metabolic Confirmation

Molecular testing identified a homozygous ALDH5A1 missense variant, NM_001080.3:c.434A >G (p.Glu145Gly), which was classified as a variant of uncertain significance. The variant was not listed in ClinVar, was absent from gnomAD, and was predicted to be damaging by in silico tools. The genetics team considered the overall presentation clinically concordant with SSADHD because of the convergence of the patient’s phenotype, elevated urinary 4‐hydroxybutyric acid, characteristic MRI abnormalities, and biallelic ALDH5A1 finding. The variant was also noted to lie within a protein‐binding domain where another pathogenic missense variant has been reported [8]. The diagnosis therefore rested on combined biochemical, imaging, clinical, and molecular evidence rather than molecular classification alone [1, 2, 8].

The family was counseled that no curative treatment is currently established [1, 8] and that care is directed toward manifestations, including seizure monitoring, neurobehavioral management, school supports, and therapies. Valproate was discussed as a medication to avoid when possible because of the potential inhibition of the SSADH enzyme activity [4, 8].

### 2.4. Hospital Course and Phenomenology

During HD2–HD4, the patient developed a progressive neuropsychiatric syndrome marked by fearfulness, reduced oral intake, hallucinations, agitation, impaired attention, perseveration, and increasing behavioral disorganization. Multidisciplinary evaluation by hospital pediatrics, neurology, psychiatry, psychology, and genetics was pursued, and no standing antipsychotic medication was initially recommended while medical and neurologic etiologies were clarified.

By HD4–HD5, clinically significant symptoms were already established before repeated hydroxyzine exposure. Mental status findings included intermittent inconsolability despite parental reassurance, limited and sometimes scripted speech, suspiciousness, impulsivity, variable intense or avoidant eye contact, distractibility, impaired attention and concentration, concrete and perseverative thought process, illogical associations, limited insight, and impaired judgment. She repeatedly described seeing a “man” or “ghosts” and was at times observed responding to internal stimuli. Catatonia was considered early in the admission; however, a Bush–Francis Catatonia Rating Scale score of 9 was driven primarily by withdrawal, perseveration, echophenomena/verbigeration in the context of intellectual disability, and one autonomic parameter [9]. Core catatonic signs, including stupor, mutism, posturing, waxy flexibility, rigidity, negativism, and marked psychomotor inhibition, were not prominent. Catatonia was therefore retained in the differential diagnosis but was not favored as the primary explanation.

Hydroxyzine remained clinically relevant because, after its initiation, impaired attention, severe sleep–wake disruption, inconsolability, increased startle response, fluctuating agitation, and waxing and waning behavioral organization persisted despite its intended anxiolytic and sedating effects. The formulation was not that hydroxyzine caused the entire syndrome as hallucinations, insomnia, poor intake, anxiety, and behavioral dysregulation all preceded exposure. Rather, hydroxyzine was considered a possible contributor to the persistence or worsening of the acute confusional state in a child with multiple delirium vulnerabilities, including severe sleep deprivation, constipation, malnutrition/poor intake, developmental disability, abnormal EEG findings, suspected metabolic disease, and acute hospitalization.

By HD6–HD7, the psychiatry team increasingly conceptualized the presentation as hyperactive delirium due to multiple etiologies. This formulation was based on acute deviation from baseline, impaired attention, profound sleep–wake disruption, inconsolability, increased startle response to sensory stimuli, affective lability, fluctuating behavioral organization, and variable ability to engage in developmentally appropriate activities. The patient alternated between crying, yelling, fearfulness, muttering, partial calm, and intermittent engagement. Distressing statements occurred during periods of severe dysregulation, but no organized suicidal or violent behavior was documented.

A pediatric delirium symptom checklist supported the clinical formulation, documenting acute change from baseline, impaired attention, inconsolability or listless irritability, difficulty being consoled with usual comfort measures, increased startle response, hallucinations, paranoid or delusional thoughts, and severe disruption of the day–night sleep cycle [7]. Disorientation to familiar caregivers was not consistently present, and the level of consciousness remained grossly intact when assessable. However, attention and behavioral organization were significantly impaired and fluctuating. Nonconvulsive seizures were considered, but long‐term video EEG did not capture electrographic seizures.

Hydroxyzine was discontinued on HD7 because of limited efficacy and concern for the anticholinergic burden in an actively delirious, medically vulnerable child. Quetiapine 25 mg by mouth nightly was initiated that evening, and a daytime PRN dose of 12.5 mg was administered on HD8. Melatonin and nonpharmacologic delirium interventions were continued, including caregiver presence, reorientation, sleep–wake regulation, minimization of nighttime disruption, promotion of daytime light and activity, familiar objects and activities, bowel regimen optimization, and nutritional support.

Over HD8–HD10, auditory and visual hallucinations and fluctuating distress persisted intermittently, but sleep quality improved, appetite and oral intake increased, and episodes of prolonged dysregulation became less severe. The patient gradually resumed preferred and developmentally appropriate activities, including smiling, dancing, playing with blocks, bathing, and interacting with the family. At discharge, she appeared brighter and more socially engaged, remained hyperactive but redirectable, and was not continuously internally preoccupied on examination. Although intermittent hallucinations were still reported by the family, improvement in sleep–wake organization, oral intake, behavioral regulation, and overall stability supported discharge with close outpatient follow‐up.

### 2.5. Psychosis Versus Delirium

The case was conceptualized as psychotic symptoms with superimposed hyperactive delirium rather than as a single uncomplicated psychotic disorder. The distinction is important because hallucinations and paranoia were present before hydroxyzine exposure and may reflect SSADHD‐associated psychotic symptoms, whereas the superimposed syndrome of fluctuating attention, severe sleep–wake disruption, inconsolability, increased startle, and waxing and waning behavioral organization supported delirium.

### 2.6. Adverse Drug Reaction Assessment

A retrospective Naranjo et al. [10] Adverse Drug Reaction Probability Scale assessment yielded an estimated score of 3, corresponding to a possible adverse drug reaction. The score reflected temporal association after exposure, improvement after discontinuation within a broader treatment package, and prior evidence that anticholinergic/deliriogenic medications may contribute to delirium risk [11, 12]. The score was limited by several competing causes, absence of rechallenge, absence of serum drug levels, and lack of systematically documented peripheral anticholinergic signs such as mydriasis, urinary retention, or dry mucosa. Therefore, the manuscript avoids the phrase “hydroxyzine‐induced delirium” as a definitive claim and instead uses “temporally associated with and possibly exacerbated by hydroxyzine.“

### 2.7. Outcome and Follow‐Up

The patient was discharged on HD10 with quetiapine 25 mg by mouth nightly and plans for psychiatry and genetics follow‐up. Outpatient referrals included psychology for developmental and diagnostic evaluation and occupational therapy to support the recovery of activities of daily living and feeding‐related function.

After discharge, the family used quetiapine as needed rather than consistently as a scheduled nightly medication. Across the longitudinal follow‐up, intermittent psychotic symptoms recurred during periods of heightened stress. During one severe recurrence while the family was traveling internationally, PRN quetiapine was perceived by the family as helpful enough to support stabilization and return home. Over time, the family reported that low‐dose quetiapine became less effective. Formal cognitive testing later documented a full‐scale IQ of 40, and she remained enrolled in special education services with an individualized education program. At a more recent follow‐up, she remained connected with psychiatry and primary care and was reported to be doing well with as‐needed olanzapine. No recurrent hydroxyzine exposure was reported in the available follow‐up record.

## 3. Discussion

This case is consistent with the expanding SSADHD literature, which describes a heterogeneous phenotype involving developmental delay, intellectual disability, communication impairment, sleep disturbance, anxiety, attention problems, hyperkinetic behavior, seizures, and psychiatric symptoms [1–3]. Psychotic symptoms appear to affect only a minority of patients, but published reports indicate that auditory and visual hallucinations, severe anxiety, aggression, sleep disturbance, behavioral dysregulation, and catatonia‐like features can occur in SSADHD, particularly during adolescence or young adulthood [4–6].

The case contributes to the literature in three ways. First, it reinforces that SSADHD should be considered when acute psychotic symptoms occur in a child with developmental delay, abnormal neuroimaging, abnormal EEG findings, parental consanguinity, and suggestive biochemical testing. Second, it illustrates that psychotic symptoms and delirium can coexist in a developmentally vulnerable child, requiring careful phenomenologic assessment rather than assuming that hallucinations alone define the syndrome. Third, it identifies a possible medication safety signal: hydroxyzine may have an added anticholinergic or sedating burden in a patient already at high risk for delirium.

Hydroxyzine is best interpreted as a possible exacerbating or prolonging factor, not as the proven sole cause of delirium. This interpretation is more clinically and scientifically defensible than definitive causal language because hallucinations, insomnia, poor intake, and behavioral dysregulation began before hydroxyzine. At the same time, the medication was temporally associated with persistent and clinically apparent delirium features, had limited efficacy for the core syndrome, and was discontinued because of concern for the anticholinergic burden. The subsequent improvement after discontinuation occurred together with quetiapine initiation, melatonin, bowel and nutritional support, family presence, and environmental sleep–wake interventions. These simultaneous changes prevent the attribution of improvement to any single intervention.

The proposed anticholinergic mechanism should also be framed cautiously. First‐generation H1 antihistamines are often considered potentially deliriogenic because their anticholinergic effects can worsen attention, arousal, and cognition in susceptible patients. However, the evidence linking individual medications to delirium varies by population, study design, and confounding risk factors [11, 12]. In this case, classic peripheral anticholinergic signs were not systematically documented. Therefore, the anticholinergic burden remains a plausible hypothesis rather than a demonstrated mechanism.

Quetiapine was selected at a low dose to target sleep–wake dysregulation, agitation, and psychotic symptoms while minimizing medication burden. In SSADHD and intellectual disability, antipsychotics should be used cautiously because of seizure threshold considerations, metabolic adverse effects, extrapyramidal symptoms, and rare but serious reactions such as neuroleptic malignant syndrome. Nevertheless, published SSADHD reports describe symptom improvement with selected antipsychotics in some patients [4–6]. In the present case, clinical improvement followed a cautious low‐dose quetiapine strategy embedded within multidisciplinary medical care and nonpharmacologic delirium management.

The molecular result also illustrates an important point in rare‐disease diagnosis. Although the patient’s ALDH5A1 variant was formally classified as a VUS, the diagnosis of SSADHD was clinically supported by the convergence of the phenotype, urine organic acid findings, and characteristic MRI abnormalities. In such cases, integrated interpretation by genetics and metabolic specialists is essential, and the molecular classification should be presented transparently without overstating the certainty based on sequence data alone [1, 2, 8].

## 4. Limitations

Several limitations should be emphasized. The case cannot establish that hydroxyzine caused delirium; it can only support a possible temporal and biologically plausible contribution. Major symptoms predated hydroxyzine exposure, and competing contributors included severe sleep deprivation, poor intake, constipation, malnutrition, abnormal EEG findings, hospitalization, underlying SSADHD, and co‐occurring psychotic symptoms. Delirium features were assessed clinically and retrospectively from the documentation rather than through a prospective standardized research instrument at every timepoint. No CSF studies were documented in the available records, and not all metabolic analytes were available with exact quantitative values. Finally, the ALDH5A1 variant remained formally classified as a VUS, although the overall clinical, biochemical, and imaging picture strongly supported SSADHD.

## Author Contributions

Musa Yilanli led conceptualization, patient management, clinical interpretation, and manuscript drafting. Colton Olexa contributed to the manuscript drafting, editing, and review of clinical information. Bianca Zapanta contributed to genetic and metabolic interpretation, manuscript editing, and clinical oversight.

## Funding

No external funding was related to this article.

## Disclosure

This case was previously mentioned only in abstract/poster form as part of a broader presentation on psychotic symptoms in children with SSADHD [13]. To our knowledge, it has not been published previously as a full case report. The present manuscript provides substantially expanded information, including a hospital day‐based timeline, medication administration chronology, diagnostic work‐up, psychosis‐versus‐delirium differentiation, adverse drug reaction assessment, limitations of causal inference, and longitudinal follow‐up. All authors reviewed and approved the final manuscript.

## Consent

Written informed consent for publication was obtained from the patient’s legal guardian. Patient assent was not feasible because of developmental and cognitive limitations. Identifying details have been removed or modified to protect the patient privacy.

## Conflicts of Interest

The authors declare no conflicts of interest.

## Data Availability

Data sharing is not applicable to this article, as no datasets were generated or analyzed during the current study.
